# Mammary gland tumor promotion by chronic administration of IGF1 and the insulin analogue AspB10 in the p53^R270H/+^WAPCre mouse model

**DOI:** 10.1186/s13058-015-0518-y

**Published:** 2015-02-18

**Authors:** Bas ter Braak, Christine Siezen, Ewoud N Speksnijder, Esmee Koedoot, Harry van Steeg, Daniela CF Salvatori, Bob van de Water, Jan Willem van der Laan

**Affiliations:** Division of Toxicology, Leiden Academic Centre for Drug Research, Leiden University, Einsteinweg 55, 2333 CC Leiden, the Netherlands; Centre for Health Protection, National Institute for Public Health and the Environment (RIVM), Antonie van Leeuwenhoeklaan 9, 3721 MA Bilthoven, the Netherlands; Medicines Evaluation Board (MEB), Graadt van Roggenweg 500, 3531 AH Utrecht, the Netherlands; Central Laboratory Animal Facility, Leiden University Medical Centre (LUMC), Einthovenweg 20, 2333ZC Leiden, the Netherlands

## Abstract

**Introduction:**

Insulin analogues are structurally modified molecules with altered pharmaco-kinetic and -dynamic properties compared to regular human insulin used by diabetic patients. While these compounds are tested for undesired mitogenic effects, an epidemiological discussion is ongoing regarding an association between insulin analogue therapy and increased cancer incidence, including breast cancer. Standard *in vivo* rodent carcinogenesis assays do not pick up this possible increased carcinogenic potential.

**Methods:**

Here we studied the role of insulin analogues in breast cancer development. For this we used the human relevant mammary gland specific p53^R270H/+^WAPCre mouse model. Animals received life long repeated treatment with four different insulin (−like) molecules: normal insulin, insulin glargine, insulin X10 (AspB10) or insulin-like growth factor 1 (IGF1).

**Results:**

Insulin-like molecules with strong mitogenic signaling, insulin X10 and IGF1, significantly decreased the time for tumor development. Yet, insulin glargine and normal insulin, did not significantly decrease the latency time for (mammary gland) tumor development. The majority of tumors had an epithelial to mesenchymal transition phenotype (EMT), irrespective of treatment condition. Enhanced extracellular signaling related kinase (Erk) or serine/threonine kinase (Akt) mitogenic signaling was in particular present in tumors from the insulin X10 and IGF1 treatment groups.

**Conclusions:**

These data indicate that insulin-like molecules with enhanced mitogenic signaling increase the risk of breast cancer development. Moreover, the use of a tissue specific cancer model, like the p53^R270H/+^WAPCre mouse model, is relevant to assess the intrinsic pro-carcinogenic potential of mitogenic and non-mitogenic biologicals such as insulin analogues.

**Electronic supplementary material:**

The online version of this article (doi:10.1186/s13058-015-0518-y) contains supplementary material, which is available to authorized users.

## Introduction

For two decades there has been an intensive debate about the human risk for increased cancer incidence when using insulin analogues [1]. Insulin and insulin analogues act via the insulin receptor (IR), of which there are two isoforms, IRA and IRB, and to a lesser extent via the insulin-like growth factor 1 receptor (IGF1R). An increased residence time as well as an increased binding affinity of synthetic insulin-like molecules towards IRA and especially IGF1R might affect carcinogenesis [2,3]. Downstream signaling via these insulin receptor family members occurs via distinct pathways. Upon stimulation with insulin analogues with high affinity for IRB the PI3K/Akt pathway is activated, which is related to the metabolic role of insulin [4]. In contrast, activation of the IGF1 pathway via IGF1R results in an upregulation of both the MAPK/Erk signaling cascade and an asymmetrical activation of the Akt pathway [5], which is directly related to the limited mitogenic effect of insulin. Stimulation of the IRA results in a downstream signaling cascade similar to that observed after IGF1R activation [5]. Epidemiological studies indicate a strong association between expression levels of both IGF1 and its receptor (IGF1R) and cancer initiation/progression [6,7]. This creates a situation whereby insulin analogues with increased affinity for IRA and/or IGF1R may increase the cancer hazard.

Current approaches to assess the intrinsic carcinogenic potential of insulin analogues are limited. The binding affinity of insulin analogues to both IR and IGF1R and their subsequent activation and overall mitogenic capacity are currently used as part of the risk assessment in terms of carcinogenic potential of newly developed insulin analogues. This *in vitro* assessment is limited by the variability in the cell lines, culture conditions and proliferation assays that are used [8,9]. Furthermore, kinetic parameters, such as administration, distribution, metabolism and excretion, cannot always be captured in these *in vitro* models. Some insulin analogues that are currently on the market have also been tested in two-year carcinogenicity studies in rats and/or mice [10,11]. So far only insulin X10 (also called AspB10) has been indicated to increase the tumor incidence in the chronic rodent studies and, consequently, it never reached the market [1,10,11]. While most insulin analogues, including glargine [12-14], were negative in these chronic bioassays, several epidemiological studies showed an increased breast cancer risk [15-18], which could not be observed by others [19-26]. Altogether these data stirred concerns that growth factor-like insulin analogues are potential non-genotoxic carcinogens [27].

Genetically engineered mouse (GEM) cancer models constitute powerful, alternative methods to assess the carcinogenetic potential of non-genotoxic compounds [28]. This, in particular, involves GEMs with constitutive or conditional tissue specific deletion of tumor suppressor genes [29]. We have described a mammary gland specific dominant negative mutated p53 mouse model, p53^R270H/+^WAPCre [30]. The model is based on a point mutation corresponding to the p53 mutated hotspot p53.R273H in the human *Li Fraumeni* cancer syndrome. Mutant p53 is only expressed when Cre recombinase is induced by the whey acidic protein promoter (WAP) in the mammary gland. This leads to spontaneous mammary gland tumor formation initiated within a year.

Here we used the p53^R270H/+^WAPCre model to evaluate the carcinogenic potential of several insulin (like) molecules: insulin NPH, insulin glargine, insulin X10 and IGF1. We demonstrated that chronic exposure to insulin X10 and IGF1 significantly promotes mammary gland tumor development, while glargine and insulin do not. Yet, glargine-related tumors do have a different pro-mitogenic signaling that is distinct from control and insulin treated mice, and more reminiscent of insulin X10 and IGF1.

## Methods

### The p53^R270H/+^WAPCre mouse model

Heterozygous p53.R270H as well as the WAPCre mice were backcrossed with FVB mice over 15 times to yield a >99.99% FVB genetic background. Heterozygous conditional p53.R270H mice, >8-week-old, were crossed to transgenic WAPCre mice of the same age to generate p53^R270H/+^WAPCre mice [30,31]. The mammary gland specific Cre recombinase splices out the intronic floxed stop cassette of the p53.R270H allele that eventually would lead to spontaneous mammary gland tumor formation of one-year-old p53^R270H/+^WAPCre female mice. A high expression WAP promoter was used [32], which is already active in the mammary gland of non-pregnant, non-lactating virgin mice. Therefore, we used only nulliparous mice in this experiment. A PCR/digestion based assay was used for genotyping using the same primers as previously described [30] and subsequent restriction analysis using Hsp92II. The presence of the R270H mutation leads to digestion of the 486 bp p53 PCR amplicon into a 269 and 217 bp product. The presence of Cre recombinase was verified by a 676 bp amplicon [30]. All mice were fed *ad libitum* with RM1 diet (SDS, technilab-BMI, Someren, Holland).

### Preparation of insulin, insulin analogues and IGF1 injection solutions

The treatments included: insulin NPH (Insuman Basal, Sanofi Aventis, Gouda, The Netherlands), insulin glargine (Lantus, Sanofi Aventis), insulin X10 (AspB10, Novo Nordisk, Alphen aan den Rijn, The Netherlands) and IGF1 (Increlex, Ipsen, Hoofddorp, The Netherlands). All compounds were dissolved in their original vehicle solutions: glargine (glycerol 0.2 mol/L, m-cresol 0.025 mol/L, ZnCl2 0.0002 mol/L adjusted to pH 4.0), insulin (glycerol 0.2 mol/L, NaH2PO4 0.00135 mol/L, phenol 0.0063 mol/L, m-cresol 0.0138 mol/L, ZnCl2 0.0001 mol/L adjusted to pH 7.4), X10 (glycerol 0.2 mol/L, phenol 0.0063 mol/L, m-cresol 0.0138 mol/L, ZnCl2 0.0001 mol/L adjusted to pH 7.4) and IGF1 (benzyl alcohol 0.083 mol/L, sodium chloride 0.1 mol/L, polysorbate 20 0.0016 mol/L, acetic acid 0.0072 mol/L, sodium acetate 0.05 mol/L adjusted to pH 5.4).

### Experimental set-up

The experimental setup of the studies was examined and approved by the institute’s Ethical Committee on Animal Experimentation, in accordance with national and European legislation.

In a first panel of short term experiments, the maximal pharmalogical dose (MPD) was determined for each compound in our mouse model. In a 10 hour experiment (n = 54) the glucose drop was measured (Freestyle light, 70812–70, Abbott, Olst, The Netherlands) sequentially every hour after a single subcutaneous injection with insulin NPH, glargine, regular insulin, X10 or IGF1 (Additional file 1: Figure S1). A wide concentration range based on the literature was evaluated: insulin NPH and insulin glargine (25 nmol/kg to 125 nmol/kg); insulin X10 (480 nmol/kg to 1,800 nmol/kg); and IGF1 (5 mg/kg to 15 mg/kg) [12]. In a subsequent experiment (n = 52) the effects of frequent injections were determined (Additional file 1: Figure S2). Besides blood glucose levels, the weight and overall well-being were determined during one month of daily injections.

Based on the above data, the long-term exposure experiment was designed in which the mice were injected with 50% and 80% of the MPD according to Additional file 2: Table S1. Once the female p53^R270H/+^WAPCre mice were about eight-weeks old, they were randomly distributed in the dose groups using the program ‘randomice’. Mice were weighed every week, and a standard injection solution per compound/concentration was made to ensure an injection volume in the range of 60 to 130 uL. To avoid adverse reactions at the injection site due to frequent injection, three subcutaneous injection sites were used: neck, upper back and lower back. Mice were injected every other day for up to 67 weeks and palpated for tumors twice a week. A typical mammary gland tumor could be detected once it had a volume of about 8 mm^3^. Dimensions were noted to monitor tumor growth. Once the tumor reached a volume of about 1 cm^3^, the mouse was sacrificed and dissected. Tumors from other origins were generally difficult to palpate, so other fitness markers (weight loss, skin condition, motility, and so on) were used to decide when to sacrifice an animal.

### Histopathology and immunofluoresence

When mice were destined for sacrifice based on tumor size or other markers, they were euthanized one day after the last injection, blood was collected and serum was extracted (mini collect, Greiner Bio-one B.V., Alphen aan de Rijn, The Netherlands) according to the manufacturer’s protocol. A quarter of the tumor, liver, lung, pancreas, kidney and spleen were fixed in a neutral aqueous phosphate buffered 4% solution of formaldehyde (Klinipath/VWR, Duiven, The Netherlands) for 24 hours and stored in 70% EtOH. These tissues were embedded in paraffin wax, sectioned at 5 μm, and stained with hematoxylin and eosin (H & E) for histopathologic evaluation. Immunofluorescence (IF) was performed on twenty representative tumors (based on the H & E characterization) from the different tumor types. Citrate buffer was used for antigen retrieval, blocking was performed in 10% normal goat serum (NGS). Antibodies were diluted (1:10 to 1:800) in 1% NGS buffer. Antibodies used were smooth muscle actin (A2547, Sigma-Aldrich, Zwijndrecht, The Netherlands), cytokeratin 5 (PRB-160P, Covance, BioLegend, London, UK), cytokeratin 8 (rdi-pro61038, Fitzgerald/Bioconnect, Huissen, The Netherlands), E-cadherin (610181, BD Transduction, Breda, The Netherlands). Fluorescently labelled secondary antibodies were from Jackson Laboratories, Suffolk, UK. Images were made on a Nikon Eclipse TS100 microscope. The histopathology was based on analysis of the macroscopic tumors. The epithelial and mesenchymal cell distributions were estimated by a pathologist on morphological characteristics of these cell types.

### Western blotting

A quarter of the tumor was snap frozen in liquid nitrogen, stored at −80°C and used for subsequent protein expression profiling. A tumor piece was ground and lysed with 300 μL tumor lysis buffer (20 mM Tris–HCl, pH 7.4, 137 mM NaCl, 2 mM ethylenediaminetetraacetic acid (EDTA), 1% Triton, 10% glycerol, 1:100 protease inhibitor cocktail) for 30 minutes at 4°C. After centrifugation (10 minutes, 10,000 rpm), supernatant was collected and the protein concentration was determined (BCA TM Protein Assay Kit; Thermo Scientific, Rockford, IL, USA). The Western blotting procedures and all antibodies were identical to [8]. In addition, antibodies targeting Her2 (Cell Signaling Technology, Danvers, MA, USA), β-actin, GAPDH, ER-α and EGFR, (Santa Cruz Biotechnology, Santa Cruz, CA, USA) were used. The same endogenous control (EC) sample consisting of a mix of 30 random mammary gland tumor samples was used on each blot.

### Statistical analysis

Statistical analyses of (mammary gland) tumor-free survival curves included calculation of censored Kaplan-Meijer distribution of survival of two different treatment groups and comparison by a two-sided log-rank test using Graphpad Prism version 4.00 software. The same software was used to determine *P*-values over the mean weight, percent of epithelial cells and average number of mammary gland tumors using the two-sided t-test. For the correlation plots linear regression was applied.

## Results

### Insulin analogue treatment induces a metabolic and, at high concentrations, a toxic response

We first systematically determined the doses at which each insulin analogue induced a glucose drop (Additional file 1: Figure S1). Two hours after the injection the maximal (approximately 60%) blood glucose decrease was observed (from approximately 6 to approximately 2 mmol/L) which was followed by a glucose recovery, depending on the insulin dose. Mice injected with short-acting insulin forms (X10 and regular insulin) required more time to recover. Next, a dose dependent positive weight effect was observed during one month daily treatment for most compounds; only the highest dose of insulin NPH and IGF1 caused a weight loss compared to control (Additional file 1: Figure S2). Despite this evident adverse effect, no mortality of mice was observed so this dose was referred to as the MPD.

### Insulin X10 and IGF1 treatment of p53^R270H/+^WAPCre female mice increases body weight

**Figure 1:**
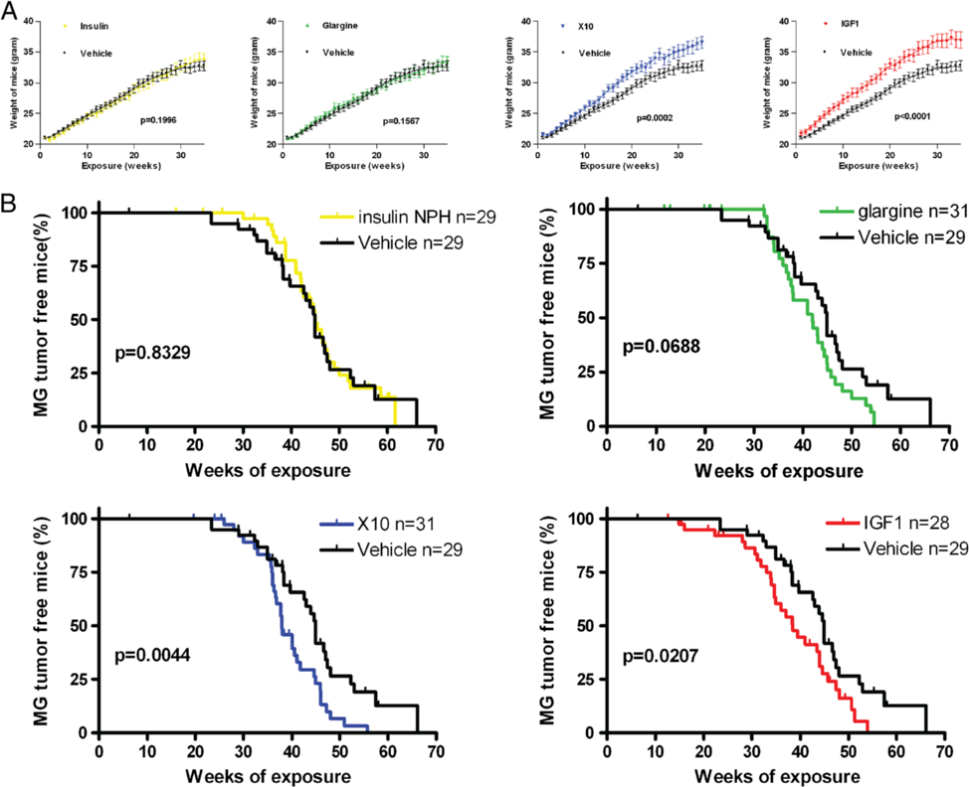
**Effect of insulin analogues on mammary gland tumor development in p53** ^**R270H/+**^ **WAPCre female mice. A)** Upper graphs represent the average weight of the mice in the indicated groups during the first 35 weeks of the experiment. Statistical analysis was determined with an unpaired t-test. **B)** Effect of different compounds on mammary gland tumor free survival shown as in Kaplan Meier curves. Statistical analysis for the survival curves was determined with the log rank test.

We then performed a life-long repeated dosing of 10-week old p53^R270H/+^WAPCre female mice with all four insulin analogues as well as the controls. We carefully monitored the weight until 35 weeks, a point in time at which the first animals developed mammary tumors (Figure 1A, top panel). X10 and IGF1 induced the strongest weight gain. In contrast, insulin and glargine did not affect the weight compared to the vehicle treated animals.

### Insulin X10 and IGF1 treatment significantly decreases the latency time for mammary gland tumor development

Next, we further followed mammary gland tumor formation. In total, 100% of the mice that remained part of the experiment developed tumors, 83% of which were mammary gland tumors with an average latency time of 40 weeks (Table 1).

**Table 1:** **Results overview of p53** ^**R270H/+**^ **WAPCre model subcutaneously injected with growth factors**

**Tested variable**	**Insulin**	**Glargine**	**X10**	**IGF1**	**Vehicle**
Number of mice analyzed	40	40	40	40	38
Mortality	6/40 (15%)	3/40 (8%)	5/40 (13%)	4/40 (10%)	1/38 (3%)
Mean weight of mice (grams)^a^	30 ± 0.5	30 ± 0.5	32 ± 0.6	34 ± 0.7	29 ± 0.4
*P*-value^b^	0.1996	0.1567	0.0002	<0.0001	NA
Tumor-bearing mice	34/34 (100%)	37/37 (100%)	35/35 (100%)	36/36 (100%)	37/37 (100%)
Mean tumor latency-time (weeks)^c^	44.9	38.0	37.9	36.0	42.6
*P*-value^d^	0.1463	0.1448	0.0707	0.0549	NA
Mammary gland tumor	29/34 (85%)	31/37 (84%)	31/35 (89%)	28/36 (78%)	29/37 (78%)
Carcinocarcoma	24/29 (83%)	25/31 (81%)	26/31 (84%)	25/28 (89%)	23/29 (79%)
EMT status (% epithelial)	20.8 ± 4.89	27.5 ± 4.83	31.2 ± 4.01	18.8 ± 4.71	15.71 ± 3.80
*P*-value^b^	0.2170	0.0672	0.0086	0.6251	NA
Carcinoma	5/29 (17%)	5/31 (16%)	5/31 (16%)	3/28 (11%)	6/29 (21%)
Adenoma	0/29	1/31 (3%)	0/31	0/28	0/29
Mean MG tumor latency-time (weeks)^c^	45.0	41.9	37.9	38.4	44.9
*P*-value^d^	0.8329	0.0688	0.0044	0.0207	NA
Mean MG tumor doubling time (days)	1.75 ± 0.11	1.87 ± 0.21	1.83 ± 0.19	1.53 ± 0.12	1.470 ± 0.14
*P*-value^b^	0.1375	0.1305	0.1632	0.7594	NA
Mice with multiple MG tumors	10/34 (29%)	16/37 (43%)	18/35 (51%)	16/36 (44%)	13/37 (35%)
Average number of MG tumors	1.50 ± 0.16	2.00 ± 0.24	1.82 ± 0.16	2.07 ± 0.23	1.64 ± 0.15
*P*-value^b^	0.5289	0.2371	0.4337	0.1346	NA

^a^Mean weight depicted as average weight of mice from start of injections (mice are ± 8 wks) until mice are sacrificed; ^b^mean latency time is depicted as number of weeks after start of the subcutaneous injections; ^c^ *P*-values determined by comparing the Kaplan-Meijer curves of compounds with vehicle; ^d^ *P*-values determined by comparing compounds with vehicle, unpaired t-test. EMT, epithelial to mesenchymal transition; MG, mammary gland.

Subcutaneous injections of vehicle 1 and vehicle 2 did not cause a significant difference in tumor latency time compared to untreated animals (*P* = 0.7194, data not shown) and were further combined in one control ‘vehicle’ group. Subcutaneous injection of the vehicle did not significantly affect the tumor latency time compared to untreated animals (*P* = 0.8476, data not shown). Each insulin analogue was dosed at 50% and 80% of the MPD. None of the different compounds showed a difference between the two doses (insulin *P* = 0.1839, glargine *P* = 0.1447, X10 *P* = 0.1619, IGF1 *P* = 0.6064; data not shown). Therefore, we also combined the two dose groups per compound. The tumor growth rate was next determined by calculating the tumor doubling time in the exponential part of the Gompertz equation curve as in [33]. All tumors showed a similar doubling time (1.44 to 1.78 day^−1^), independent of treatment conditions (Table 1).

The overall mammary gland tumor latency time (MTLT) for control mice was 44.9 weeks. IGF1 and insulin X10 caused a significantly decreased mean MTLT of 6.5 and 7.0 weeks respectively, compared to the vehicle treatment (Figure 1B; MTLT decreases from 44.9 weeks for control to 38.4 weeks (*P* = 0.0207) and 37.9 weeks (*P* = 0.0044) for IGF1 and X10, respectively). Glargine injections led to a tumor latency time reduction of three weeks, although this was not significantly different (*P* = 0.0688). No effect on MTLT was observed for treatment with human insulin (*P* = 0.8329). A total of 41% of the mice developed multiple mammary gland tumors.

### Different insulin analogues do not affect mammary gland tumor type

**Figure 2:**
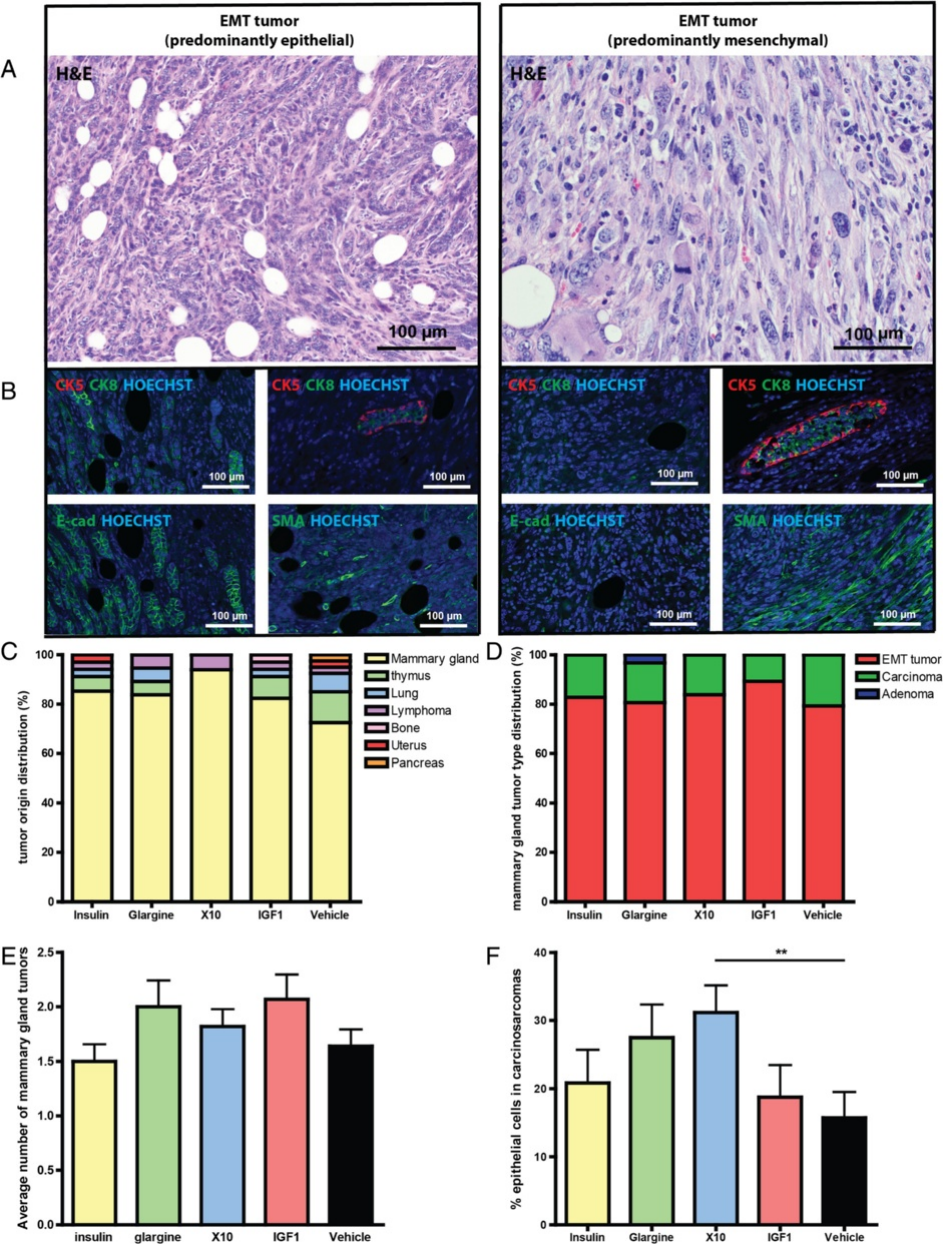
**Insulin analogues induce primarily EMT tumors in p53** ^**R270H/+**^ **WAPCre female mice. A)** H & E images of representative EMT tumors with predominantly epithelial cells (left panel) and an EMT tumor with predominantly mesenchymal cells (right panel). **B)** Immunofluoresent images of CK5/8, area with normal mammary duct on the same slide that serves as control for CK5/CK8 staining, E-cadherin and smooth muscle actin. **C)** Tumor type distribution per treatment. **D)** Mammary gland tumor type distribution per treatment. **E)** Average number of mammary gland tumors. **F)** Percentage of epithelial cells in the EMT tumors over the different treatment groups. EMT, epithelial to mesenchymal transition.

All mammary proliferative lesions were next classified according to the criteria described by the Annapolis Pathology Panel (Figure 2A) [34]. Irrespective of treatment condition, the majority (123/148, 83%) of mammary gland tumors was diagnosed as EMT (epithelial to mesenchymal transition) tumor, 16% (24/148) as carcinoma and 1% (1/148) as adenoma (Table 1).

EMT tumors with a clearly distinguishable histological epithelial component also showed clear E-cadherin staining, whereas EMT tumors that predominantly consist of mesenchymal cells showed high levels of smooth muscle actin (SMA) (Figure 2B). In total, 31 mice developed other tumors: ten thymic lymphoma, six lung adenocarcinomas, and a small number of lymphomas, bone, uterus or pancreas tumors (Figure 2C). No correlation between the prevalence of non-mammary gland tumors and treatment could be detected. Typically lymphomas developed significantly earlier (independent of treatment) compared to mammary gland EMT tumors and carcinomas (30.8 weeks versus 41.4 and 39.1 weeks, respectively).

There was no clear correlation between treatment and tumor spectrum (Figure 2D). EMT tumors were previously described as carcinosarcomas, and these terms can be used as synonyms [35]. Three-dimensional culturing of isolated cells from these primary tumors resulted in an invasive morphology, one of the characteristics of an EMT tumor (data not shown). The EMT-phenotype tumor was considered morphologically significant in the presence of epithelial structures displaying loss and disruption of basement membrane and associated with epithelial-to-spindle cell morphology transition and gradual blending or invasion into the surrounding stroma [36]. Most mammary tumors showed invasion of the surrounding fat pad. Variable-sized intratumoral areas of necrosis were also present, often accompanied by a desmoplastic reaction and severe infiltration of inflammatory cells composed mostly of neutrophils and reactive macrophages.

Some mice developed more than one mammary gland (MG) tumor. There appears to be a trend in the incidence of increased multiple MG tumors for glargine, X10 and IGF1, compared to vehicle treatment, but none of these treatments reached significance (Figure 2E).

We also assessed the percentages of epithelial and mesenchymal cells. Only X10 treatment induced significantly (*P* = 0.0086) more EMT tumors with a higher epithelial prevalence (Figure 2 F). To further assess the origin from either the basal or the luminal part of the MG ducts, the expression of CK5 and CK8 was evaluated, respectively. Based on expert judgment of the pathologist we have screened a subset of twenty tumors, equally distributed over the treatment groups. From these tumors only one was stained positive for CK5 and negative for CK8, all other tumors showed clear CK8 staining and lack of CK5 indicating that the majority of the tumors likely originated from the luminal part of the MG. A representative immunofluorescent picture of a luminal tumor and the only basal tumor that we found can be found in Additional file 1: Figure S3. No correlation was detected between the different treatment conditions or with tumor histology.

### Molecular profiling of IR and IGF1R signaling pathways in insulin analogue induced mammary gland tumors

**Figure 3:**
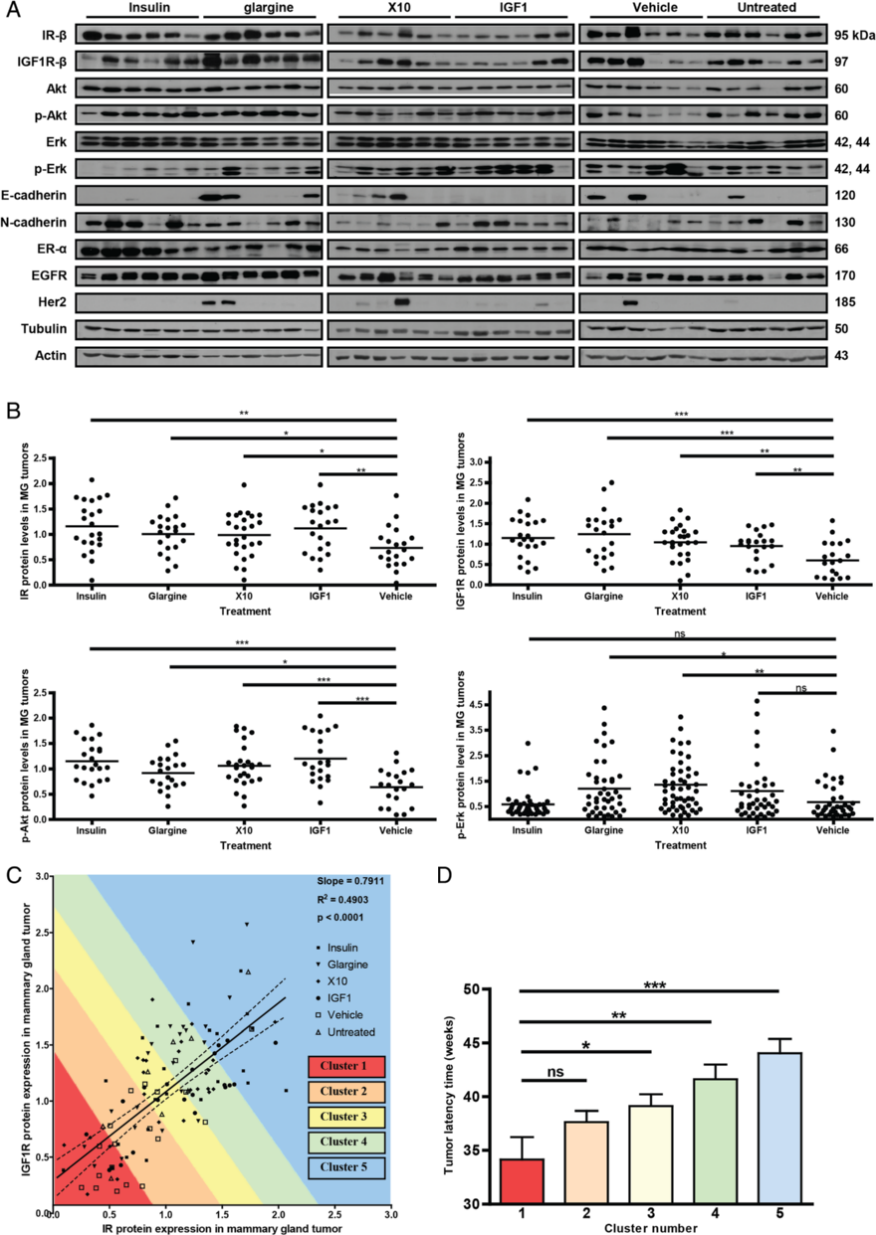
**Molecular profiling of IR and IGF1R signaling in insulin analogue derived mammary gland tumors.** Tumor protein levels of critical mammary gland tumor related receptors (IR, IGF1R, ER, EGFR, Her2) and downstream signaling pathways (Erk, phospho-Erk, Akt, phospho-Akt) as well as epithelial differentiation markers (N-cadherin and E-cadherin) were determined by quantitative Western blotting of all primary mammary gland tumors (n = 148). **A)** A small subset of the Western blot data (n = 36) is shown which are representative for the quality of the blots. **B)** The quantitative IR, IGF1R, p-Akt and p-Erk levels of all primary EMT tumors are presented in dot-plots (n = 148). **C)** The IR versus IGF1R protein levels are plotted, five clusters are defined. **D)** The mean tumor latency time has been determined of each cluster. Error bars represent SEM, ns = *P* >0.05, * = *P* <0.05, ** = *P* <0.01, *** = *P* <0.001. EGFR, epidermal growth factor receptor; EMT, epithelial to mesenchymal transition; ER, estrogen receptor; IGF1R, insulin-like growth factor 1 receptor; IR, insulin receptor; SEM, standard error of the mean.

To further understand the underlying mechanism of the decreased tumor latency by IGF1 and X10, we determined the expression of proteins in the main signaling pathways associated with IR and IGF1R signaling as well proteins used in breast cancer classification. This included the quantitative analysis of IR-β, IGF1R-β, EGFR, Her2, E-cadherin, N-cadherin, Akt, p-Akt, Erk, p-Erk, ER-α, tubulin and β-actin protein levels in all primary mammary gland tumors. A small subset of the Western blot data (n = 36) is presented in Figure 3A; the quantitative analysis of Figure 3B is based on all Western blots (n = 148) shown in Additional file 1: Figure S4. Overall, only IR, IGF1R, p-Erk or p-Akt levels were significantly affected in insulin analogue treatment-related mammary tumor (Figure 3B). Interestingly, the IR and IGF1R receptor levels in tumors of compound treated animals were all significantly upregulated compared to control. Importantly, IGF1-dependent tumors not only upregulated IGF1R, but also showed a significant (*P* = 0.0066) upregulation of IR; similarly insulin treated mice demonstrated IR upregulation but also showed significantly higher (*P* = 0.0005) IGF1R levels.

Also, phosphorylated Akt was more abundant in tumors of mice treated with insulin-like molecules; especially insulin, X10 and IGF1 stimulation led to tumors with very significant higher activation of the PI3K signaling pathway. Interestingly, glargine, X10 and IGF1 but not insulin stimulation led to an increased activation of the MAPK signaling cascade in the obtained tumors. This effect was only significant for glargine (*P* = 0.0125) and X10 (*P* = 0.0018) but not for IGF1 (*P* = 0.0980) when compared to the tumors from the vehicle-treated mice.

We also compared the association between IR and IGF1R expression (Figure 3C). Overall there seemed to be a direct linear correlation between IR and IGF1R, suggesting interdependency. To evaluate the relationship between IR and IGF1R levels and tumor latency, we separated the entire group of tumors into five equal clusters each representing 20% of the tumors based on right-angled distribution on the linear regression curve. For each cluster the mean latency time was determined irrespective of treatment condition (Figure 3D), but only involving insulin, glargine, X10 and IGF1 conditions. Intriguingly, a long latency time and, thus, a long exposure to the insulin-like molecules, is associated with increased insulin- and IGF1-receptor levels. While the tumors of all control animals showed a strong correlation between IR and IGF1R expression levels, albeit at a lower expression level (see Additional file 1: Figure S4 and Figure S3B), no correlation was observed with either latency time or receptor levels. A similar correlation was observed between IR/p-Akt and IGF1R/p-Akt (data not shown).

### Mitogenic insulin analogue tumor formation is related to enhanced Erk and Akt activity

**Figure 4:**
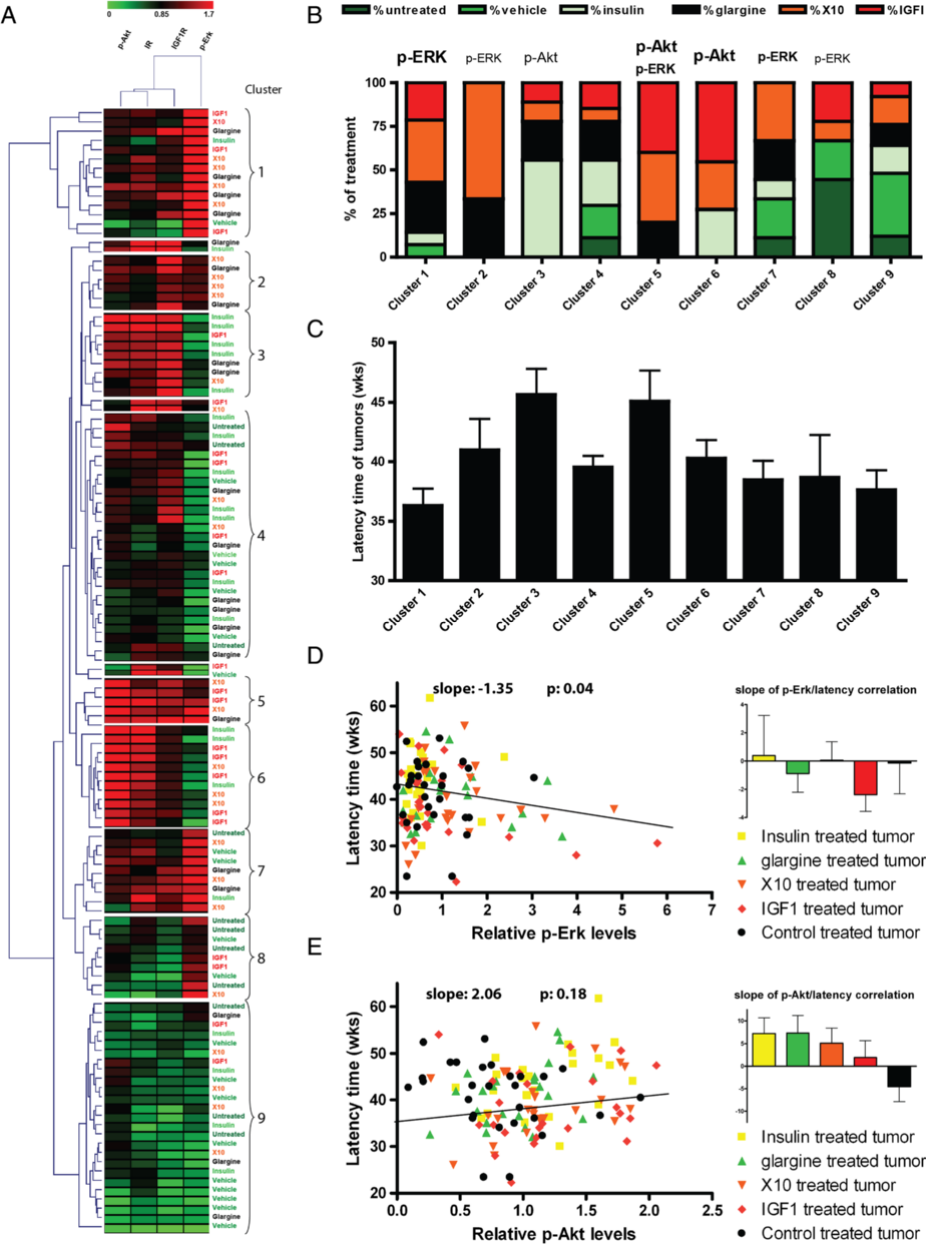
**Hierarchical clustering of insulin (analogue) tumors based on IR and IGF1R signaling components.** Quantitative expression levels of IGF1R-β, IR-β, p-Akt and p-Erk in mammary gland EMT tumors of all treatment groups were clustered using Euclidean distance and average linkage **(A)**. Distinct clusters appeared in which the treatment groups are not equally distributed. In protein clusters 1, 2, 5 and 6, the highly mitogenic treatment groups (IGF1 and X10) are overrepresented. In graphs B and C the distribution of several parameters of the clusters are shown. **B)** Treatment group distribution. **C)** Latency time per cluster, in which it became apparent that the cluster with the highest p-Erk levels has the shortest tumor latency time. In the last two graphs the correlation between latency time and p-Erk **(D)** or latency time and p-Akt **(E)** is presented. Interestingly, high p-Akt levels are positively correlated with latency time, but only in the compound treatment groups and not in the vehicle treated animals. EMT, epithelial to mesenchymal transition; IGF1R, insulin-like growth factor 1 receptor; IR, insulin receptor.

Since the insulin analogue treatment apparently affected and directed the composition and the activity of signaling machinery in the MG tumors, we next determined whether specific subgroups of profiles existed and whether these would cluster with particular insulin analogues. We focused on those signaling components that were significantly different between control and insulin-like molecule treatment (that is, IR, IGF1R, p-Erk and p-Akt) and evaluated all EMT tumors (n = 123; the major tumor type irrespective of treatment condition), thereby eliminating tumor type variation. Hierarchical clustering of the quantitative signaling data separated in nine clusters (Figure 4A and B). Five clusters showed increased p-Erk level as a dominating signaling pathway: clusters 1, 5 and 7 represented high p-Erk which was dominated by IGF1, X10 and glargine related EMT tumors (together 78%, n = 22 tumors); clusters 2 and 8 showed lower p-Erk with no clear enrichment (n = 15 tumors). In addition, two clusters especially represented Akt activation: clusters 5 and 6 preferentially showed high p-Akt activity, and together these clusters demonstrated enrichment for IGF1 and X10 treatment (75%; n = 16 tumors). Importantly, there were no tumors from the control group that showed high p-Akt levels. Two large clusters (4 and 9) did not show any dominance or activation of either Erk or Akt; in these two clusters there was a slight overrepresentation of control and insulin treatment (61%; n = 31 tumors). These combined data suggest that pro-mitogenic insulin analogues drive tumor formation that is dominated by either p-Erk and/or p-Akt, although at this point we cannot exclude the activation of additional signaling pathways. We also determined the tumor latency time for each cluster (Figure 4C). Overall, clusters enriched for pro-mitogenic insulin analogues and associated enhanced mitogenic signaling, either Erk or Akt, did not significantly correlate with latency time. Yet, a tendency existed for a shorter latency time in cluster 1 which had the highest p-Erk level. Therefore, we also determined the correlation between either p-Erk or p-Akt status for any treatment condition and MG tumor latency time (Figure 4D and E). A significant negative correlation was found between p-Erk status and latency time. This effect was strongest for IGF1-induced tumors, but glargine-induced tumors also showed this trend. A weak non-significant positive correlation was found between p-Akt status and EMT tumor latency time. Interestingly, this positive effect was observed for all treatments, except for the control group.

## Discussion

In the present study, we used the p53^R270H/+^WAPCre mouse model to assess the carcinogenic potential of two insulin-like molecules, X10 (AspB10) and glargine in direct comparison to insulin NPH and IGF1. Our data indicate that the highly mitogenic compounds, insulin X10 and IGF1, which both stimulate the IGF1-receptor, significantly decreased the latency time for tumor development. This was not observed for glargine and insulin NPH. Moreover, we demonstrated that tumors derived after mitogenic insulin analogue treatment induce mammary gland tumors with enhanced intracellular signaling through either the Erk or Akt pathway, which was not observed in control animals. Our data indicate that the p53^R270H/+^WAPCre mouse model is sensitive to evaluate the intrinsic higher mitogenic potential of insulin-like compounds and the associated contribution to cancer development. Interestingly, while this mouse tumor model largely gives rise to EMT mammary gland tumors irrespective of insulin treatment conditions, the mitogenic insulin-like molecules drive the formation of tumors with enhanced key mitogenic signaling activity.

X10 as well as IGF1 significantly decreased tumor latency time. These two compounds also induced a significant weight increase. However, there is no overall correlation between tumor latency time and mouse weight at tumor detection date (Additional file 1: Figure S5), indicating that chronic treatment of X10 and IGF1 affects tumor latency directly by mitogenic signaling rather than indirectly by obesity.

Similar to insulin NPH, chronic exposure to insulin glargine did not significantly affect tumor latency time compared to the vehicle treatment, although tumors developed slightly earlier in the glargine treated animals. These glargine data are in line with a previous report by Stammberger *et al*. in which a lifelong exposure of glargine did not show any difference in the incidence of mammary tumors reported in both mice and rats when comparing with the NaCl, vehicle-control, or the NPH insulin treated groups in wild type mice and rats [13]. When conducting a pathology study on the different tumors, we found no difference between insulin NPH versus glargine induced tumors. These histopathology results are in agreement with a study performed by Besic *et al*., in which a clinical and histopathological screening was performed on breast carcinomas of diabetic patients who were either on a glargine or other insulin (analogue) therapy [37].

We previously showed in *in vitro* models that insulin IGF1, X10 and glargine can strongly activate the IGF1R-mediated signaling, but that only IGF1 and X10 have an increased mitogenic potential [8]. Insulin glargine did not significantly enhance cell proliferation, which was largely explained by the rapid metabolism of glargine, preventing a sustained activation of IGF1R-mediated mitogenic signaling [8]. These insulin-like compounds have been tested for carcinogenic side-effects in wild type mice and rats [10,11,38], but to our knowledge they have never been tested in sensitive humanized *in vivo* models. Our current *in vivo* findings are in agreement with these *in vitro* observations. Yet, despite the fact that insulin glargine did not significantly enhance MG tumor development, many insulin glargine tumors also demonstrated enhanced Erk and Akt activity, which was hardly observed under control conditions. This suggests that insulin glargine is not inert and may affect the intracellular signaling in the developing tumor.

The majority of the obtained tumors in our p53^R270H/+^WAPCre mouse model were classified as EMT tumors; these tumors are thought to be in the epithelial to mesenchymal transition state. This type of MG tumor is not a commonly found human breast cancer subtype. Nevertheless, as in human breast cancer, the intracellular signaling varied considerably between the various tumors. In particular, a strong enhancement of Erk and Akt activity was evident in the mitogenic insulin-like molecules groups, which was hardly observed for untreated mice. This variation induced by our insulin treatment conditions probably provides a better representation of human breast cancer in general and EMT tumors in particular. Hence, a mitogenic treatment setting might provide enhanced information on human breast cancer development. Yet, given the biased formation of EMT tumors in the current study setup, further carcinogenic studies with insulin-like molecules are required with other humanized mouse MG tumor models. This could involve genetically engineered mouse models (GEMMs) with a human specific mutation in, for example, the PI3K signaling pathway [39].

Long-term administration of IGF1, X10, glargine and insulin led to tumors with significantly higher p-Akt levels compared to vehicle treated animals. This indicates that the PI3K signaling cascade is up regulated upon stimulation with the insulin-like molecules. Similarly the MAPK signaling pathway was up regulated after IGF1, X10 and glargine treatment. Interestingly, long-term stimulation with insulin did not affect the p-Erk1/2 levels in the obtained tumors.

At this moment we do not know the exact mechanism by which the more mitogenic insulin like molecules promotes MG tumor development. Since Erk and Akt activity did not *per se* coincide in the various tumors and/or relate to enhanced IGF1R levels, direct ligand-mediated activation of the Erk and Akt pathways seems unlikely. Possibly, mutations in either Erk and/or Akt pathway components are underlying the enhanced activation of these signaling molecules. In such a scenario, activation of the IGF1R and/or IR may promote the selection of the initiated cells that have incorporated mutations in Erk/Akt pathway components (for example, Ras or PI3K). This could provide a suggestion that treatment with human insulin analogues may initiate the development of mammary tumors with an altered mutational and/or signaling spectrum. More in-depth molecular analysis of the mouse tumors at the genome and proteome level is needed to further understand the underlying mechanism for the enhanced tumor formation by the IGF1 and X10 treatment conditions and their potential role in either enhanced tumor initation and/or progression. While we studied the effect of insulin analogues on MG tumor development, we cannot exclude that insulin analogues with high affinity for the IGF1R may also promote breast cancer progression, either locally or at distant sites, or modulate sensitivity to anticancer drugs. This needs further investigation and our different mouse MG tumor banks that show different expression levels of IGF1R and IR could contribute to this.

## Conclusions

The p53^R270H/+^WAPCre mouse model is a sensitive and human relevant model to test the carcinogenic properties of insulin-like molecules, as is apparent with insulin X10 and IGF1. Insulin glargine was tested in this study and did not show a significantly decreased tumor latency time compared to insulin NPH, although the MAPK-signaling pathway was upregulated as found for X10 and IGF1. As is the case in humans, rapid conversion of glargine into metabolically active metabolites M1 (and to a lesser extent M2) is likely to be the reason for the low carcinogenic potential of subcutaneous injected glargine. All in all, based on the current tumor model, the data suggest that glargine users are not facing an increased carcinogenic hazard compared to insulin NPH users. Yet, future studies in mouse models that lead to more human relevant tumors remain important to fully exclude a role for current clinically relevant insulin analoques in the development and/or progression of human breast cancer.

## Additional files

Additional file 1: Figure S1. Blood glucose levels measured in p53^R270H/+^WAPCre mice after injections with a concentration range of insulin(−like) molecules. A) insulin NPH injections, B) glargine injection, C) IGF1 injections, D) regular human insulin injections and E) X10 injections. The number 1 graphs represent the blood glucose levels of injected mice over time. The number 2 bar plots represent the area above the curve of the first blood glucose drop (first three hours). Each data point represents the average blood glucose levels of two mice. **Figure S2.** Weight measured in p53^R270H/+^WAPCre mice after injections with a concentration range of insulin(−like) molecules. A) Insulin NPH injections, B) glargine injection, C) IGF1 injections, D) regular human insulin injections and E) X10 injections. The number 1 graphs represent the weight increase in % of injected mice over time. The number 2 bar plots represent the area under the curve of the weight increase plots. **Figure S3.** Origin of tumors. Representative EMT tumor originating from myo-epithelial part (first IF image which is CK5+) or luminal part (second IF image which is CK8+) of mammary gland (MG) (n = 19). **Figure S4.** Protein expression profiles of all primary mammary gland tumors. Tumor protein levels of critical mammary gland tumor-related receptors (IR, IGF1R, ER, EGFR, Her2) and downstream signaling pathways (Erk, phospho-Erk, Akt, phosphor-Akt) as well as epithelial differentiation markers (N-cadherin and E-cadherin) were determined by quantitative Western blotting of all primary mammary gland tumors (n = 148). EC is the endogenous control, a sample that was loaded on every blot to correct for blot specific effects. **Figure S5.** No correlation between tumor latency time and weight of mice at tumor detection date. (*P* = 0.8939, best fit linear slope −0.022). Additional file 2: Table S1. Set-up of long-term exposure experiment.

## Abbreviations

bp: base pair
EC: endogenous control
EMT: epithelial to mesenchymal transition
GEM: genetically engineered mouse
IF: immunofluorescence
IGF1: insulin-like growth factor 1
IR: insulin receptor
MG: mammary gland
MPD: maximal pharmalogical dose
MTLT: mammary gland tumor latency time
NGS: normal goat serum
WAP: whey acidic protein
