# Supplementary material for: Mammary gland tumor promotion by chronic administration of IGF1 and the insulin analogue AspB10 in the p53R270H/+WAPCre mouse model
Source: Breast Cancer Res. 2015 Feb 18;17(1):14. doi: 10.1186/s13058-015-0518-y (PMC4349771; doi:10.1186/s13058-015-0518-y)
Supplement: Additional file 1: Figure S1. — Blood glucose levels measured in p53R270H/+WAPCre mice after injections with a concentration range of insulin(−like) molecules. A) insulin NPH injections, B) glargine injection, C) IGF1 injections, D) regular human insulin injections and E) X10 injections. The number 1 graphs represent the blood glucose levels of injected mice over time. The number 2 bar plots represent the area above the curve of the first blood glucose drop (first three hours). Each data point represents the average blood glucose levels of two mice. Figure S2. Weight measured in p53R270H/+WAPCre mice after injections with a concentration range of insulin(−like) molecules. A) Insulin NPH injections, B) glargine injection, C) IGF1 injections, D) regular human insulin injections and E) X10 injections. The number 1 graphs represent the weight increase in % of injected mice over time. The number 2 bar plots represent the area under the curve of the weight increase plots. Figure S3. Origin of tumors. Representative EMT tumor originating from myo-epithelial part (first IF image which is CK5+) or luminal part (second IF image which is CK8+) of mammary gland (MG) (n = 19). Figure S4. Protein expression profiles of all primary mammary gland tumors. Tumor protein levels of critical mammary gland tumor-related receptors (IR, IGF1R, ER, EGFR, Her2) and downstream signaling pathways (Erk, phospho-Erk, Akt, phosphor-Akt) as well as epithelial differentiation markers (N-cadherin and E-cadherin) were determined by quantitative Western blotting of all primary mammary gland tumors (n = 148). EC is the endogenous control, a sample that was loaded on every blot to correct for blot specific effects. Figure S5. No correlation between tumor latency time and weight of mice at tumor detection date. (P = 0.8939, best fit linear slope −0.022). [file 13058_2015_518_MOESM1_ESM.pdf]

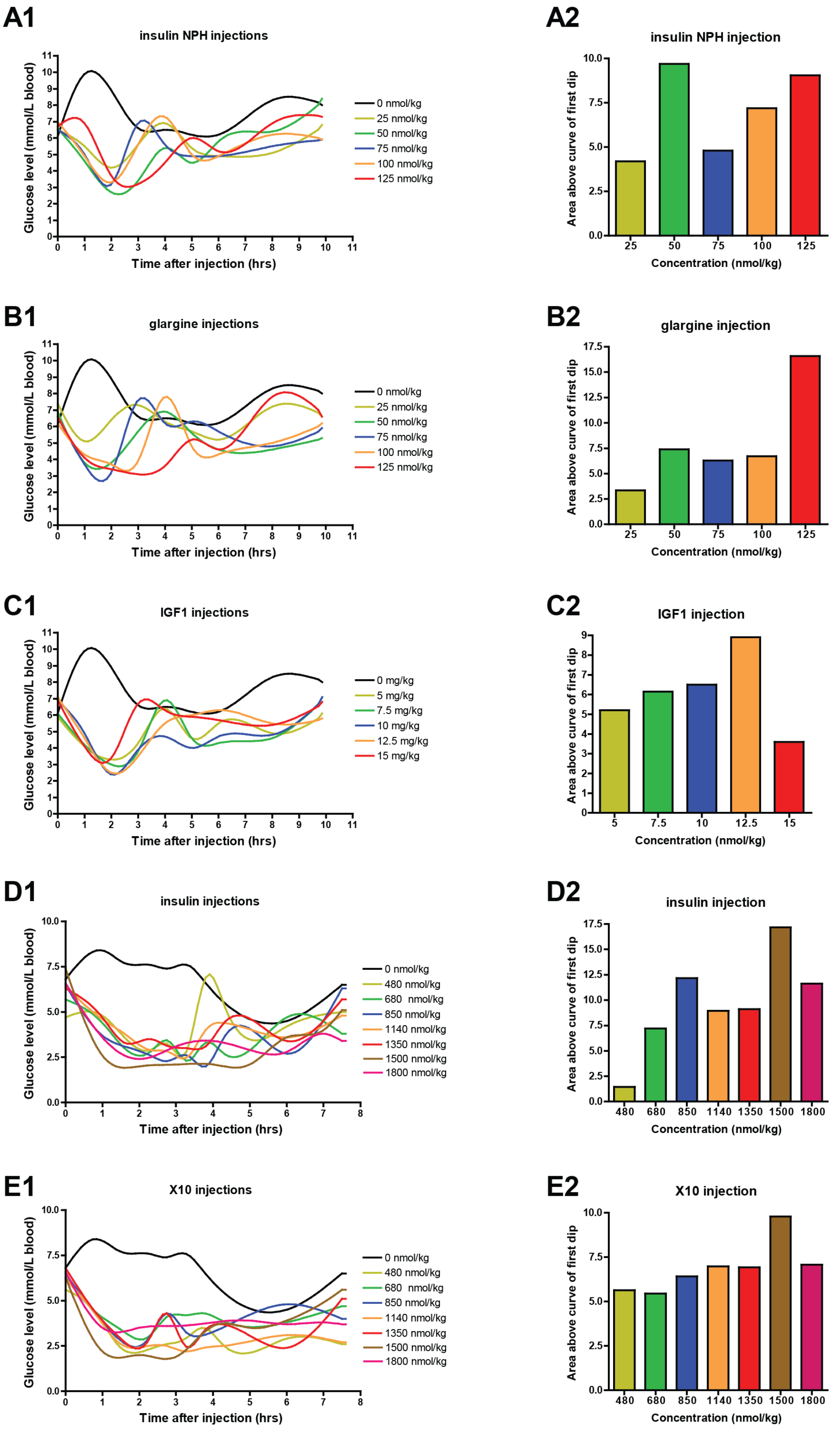

Supplemental Figure 1.

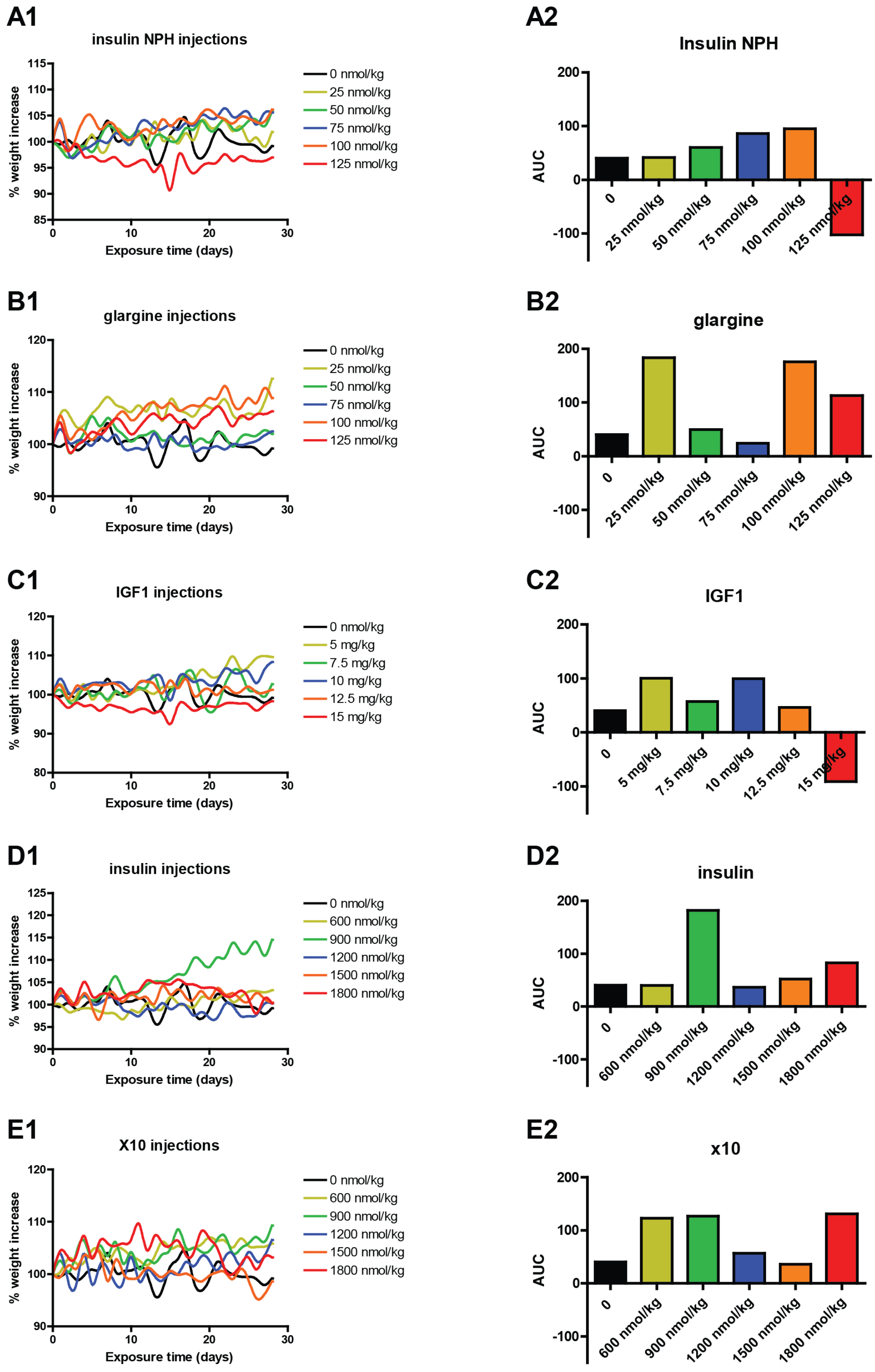

Supplemental Figure 2.

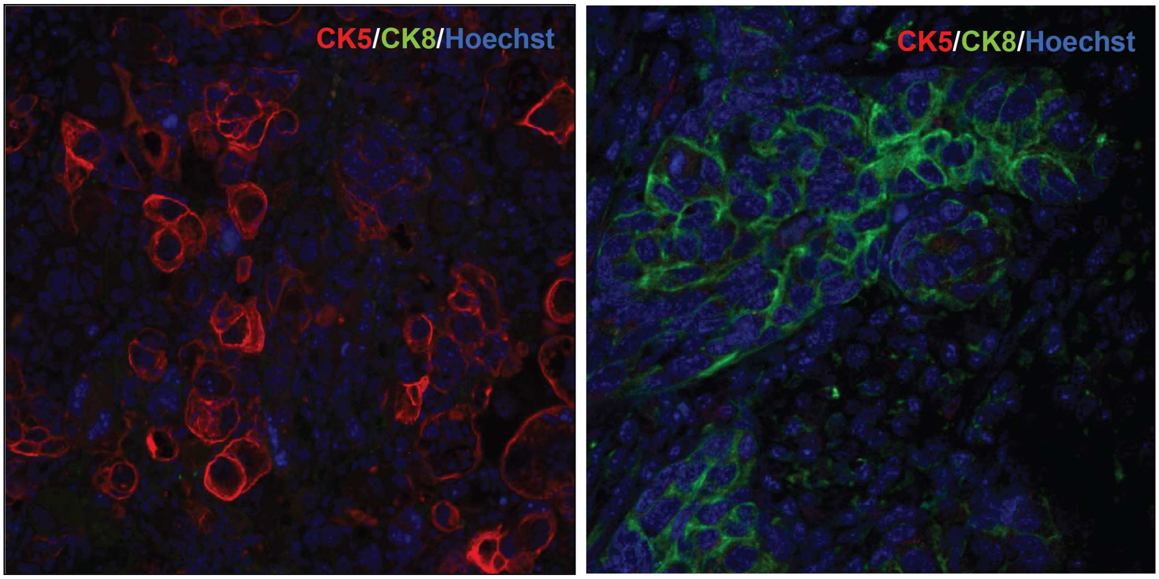

Supplemental Figure 3.

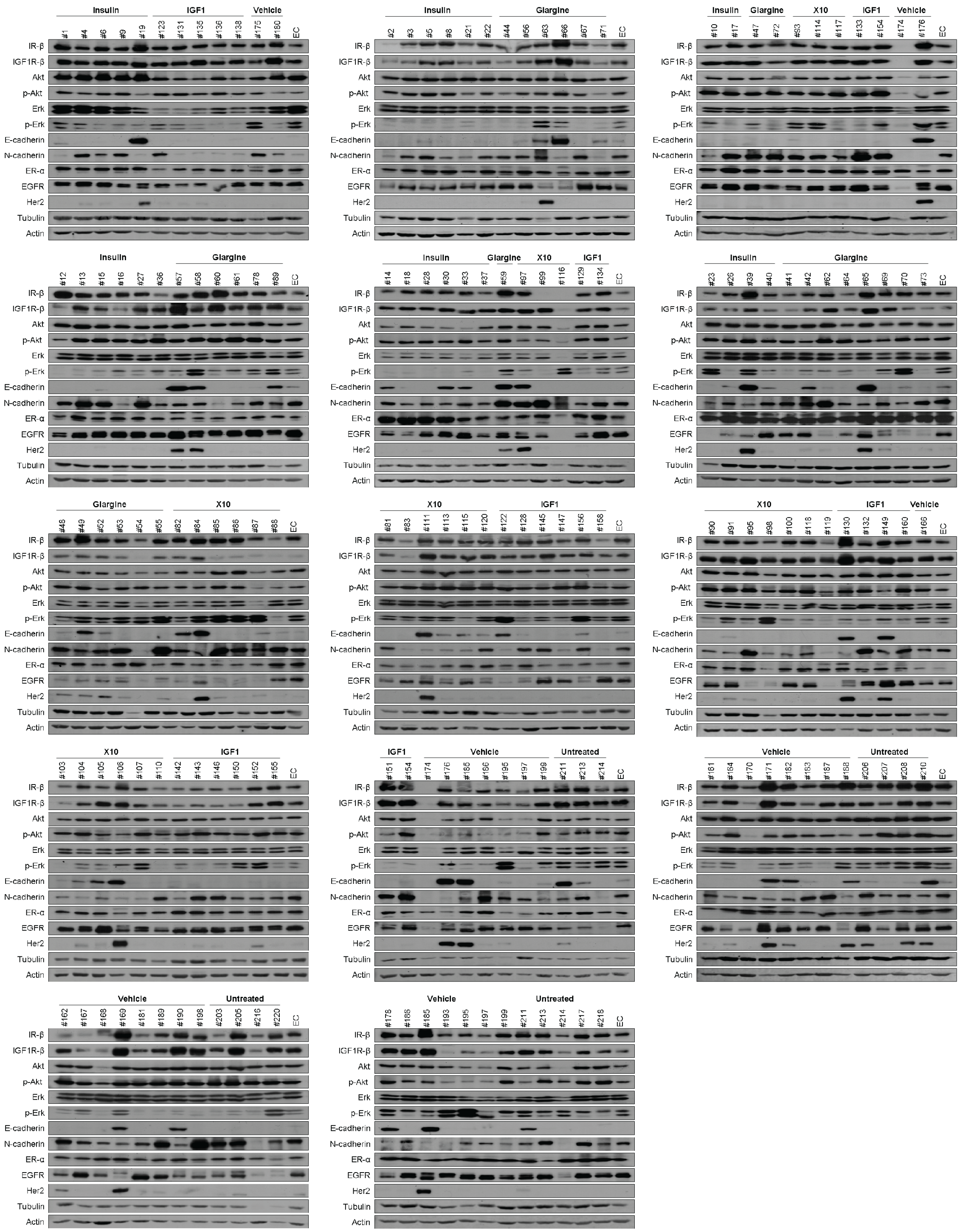

Supplemental Figure 4.

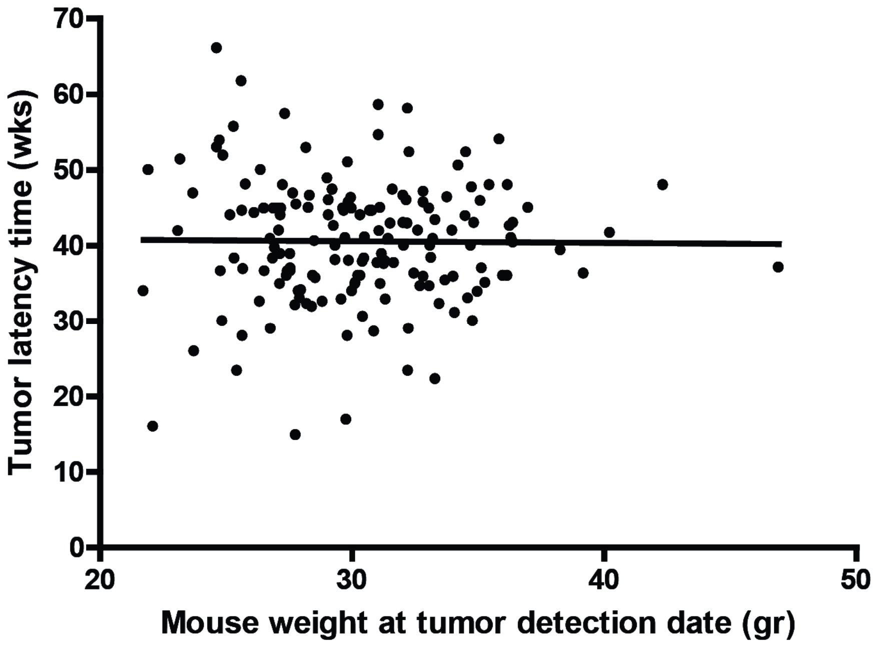

Supplemental Figure 5.
