# Supplementary material for: Mammary gland tumor promotion by chronic administration of IGF1 and the insulin analogue AspB10 in the p53R270H/+WAPCre mouse model
Source: Breast Cancer Res. 2015 Feb 18;17(1):14. doi: 10.1186/s13058-015-0518-y (PMC4349771; doi:10.1186/s13058-015-0518-y)
Supplement: Additional file 2: Table S1. — Set-up of long-term exposure experiment. [file 13058_2015_518_MOESM2_ESM.doc]

| Table S1. Set-up of long-term exposure experiment | | | | | | |
| --- | --- | --- | --- | --- | --- | --- |
| **Compound** | **Dose nmol/kg** | **Dose mg/kg** | **Dose iU/kg** | **Metabolic potential** | **Carcinog. potential** | **# of mice** |
| Insulin NPH | 75 | 0.44 | 12.5 | + | - | 20 |
|  | 100 | 0.58 | 16.7 |  |  | 20 |
| Glargine | 75 | 0.46 | 12.5 | + | ? | 20 |
|  | 100 | 0.61 | 16.7 |  |  | 20 |
| X10 | 900 |  | 150 | + | + | 20 |
|  | 1200 |  | 200 |  |  | 20 |
| IGF1 | 1307 | 10 | NA | - | + | 20 |
|  | 1634 | 12.5 | NA |  |  | 20 |
| Vehicle 1 (LANTUS) | NA |  |  | - | - | 20 |
| Vehicle 2 (BASAL) | NA |  |  | - | - | 20 |
| Untreated | NA |  |  | - | - | 20 |
